# Concatenated Modified LeNet Approach for Classifying Pneumonia Images

**DOI:** 10.3390/jpm14030328

**Published:** 2024-03-21

**Authors:** Dhayanithi Jaganathan, Sathiyabhama Balsubramaniam, Vidhushavarshini Sureshkumar, Seshathiri Dhanasekaran

**Affiliations:** 1 Department of Computer Science and Engineering, Sona College of Technology, Salem 636005, India; sathiyabhama@sonatech.ac.in; 2 Department of Computer Science and Engineering, Faculty of Engineering and Technology, SRM Institute of Science and Technology, Vadapalani Campus, Chennai 600026, India; vidhushs@srmist.edu.in; 3 Department of Computer Science, UiT The Arctic University of Norway, 9037 Tromsø, Norway

**Keywords:** pneumonia, convolution neural network, modified LeNet, classification, ReLU

## Abstract

Pneumonia remains a critical health concern worldwide, necessitating efficient diagnostic tools to enhance patient care. This research proposes a concatenated modified LeNet classifier to classify pneumonia images accurately. The model leverages deep learning techniques to improve the diagnosis of Pneumonia, leading to more effective and timely treatment. Our modified LeNet architecture incorporates a revised Rectified Linear Unit (ReLU) activation function. This enhancement aims to boost the discriminative capacity of the features learned by the model. Furthermore, we integrate batch normalization to stabilize the training process and enhance performance within smaller, less complex, CNN architectures like LeNet. Batch normalization addresses internal covariate shift, a phenomenon where the distribution of activations within a network alter during training. These modifications help to prevent overfitting and decrease computational time. A comprehensive dataset is used to evaluate the model’s performance, and the model is benchmarked against relevant deep-learning models. The results demonstrate a high recognition rate, with an accuracy of 96% in pneumonia image recognition. This research suggests that the Concatenated Modified LeNet classifier has the potential to be a highly useful tool for medical professionals in the diagnosis of pneumonia. By offering accurate and efficient image classification, our model could contribute to improved treatment decisions and patient outcomes.

## 1. Introduction

Pneumonia, a severe lung infection, can become fatal if left untreated. This wide-spread illness arises from diverse microorganisms like bacteria, viruses, and fungi. The word itself originates from the Greek term “Neuman” meaning the lungs, establishing its close connection to lung-related disorders. In medical terms, pneumonia signifies inflammation within the lung tissue (parenchyma) [1].

Nevertheless, pneumonia can also result from factors such as inhaling food particles or exposure to harmful chemicals. Pneumonia typically arises from an infection, and it is characterized by inflammation induced by pathogens. This inflammation leads to the growth of pus in the lung’s alveoli, thereby hindering the exchange of carbon dioxide (CO_2_) and oxygen (O_2_) between the blood and the lungs. This, in turn, makes it difficult for individuals with pneumonia to breathe. Common signs of pneumonia include dyspnea, fever, tussis, and thoracic pain, among others. Certain individuals are at a higher risk of contracting pneumonia, including elderly persons, children, and individuals with underlying health conditions such as HIV/AIDS, diabetes mellitus, chronic diseases, cardiovascular ailments, cancer, and hepatic diseases [2,3,4,5]. The hybrid optimization algorithm was proposed by Vidhushavarshini Suresh et al. [6] for feature selection in a thyroid disease classifier, utilizing rough type-2 fuzzy support vector machine. The hybrid algorithm combined firefly and butterfly optimization algorithms to select top-n features. The proposed HFBO-RT2FSVM model achieves high accuracy (99.28%), specificity (98%), and sensitivity (99.2%). Comparative analysis against benchmark methods demonstrated a significant improvement in disease identification [6].

### 1.1. Pneumonia Imaging Modalities

**Figure 1:**
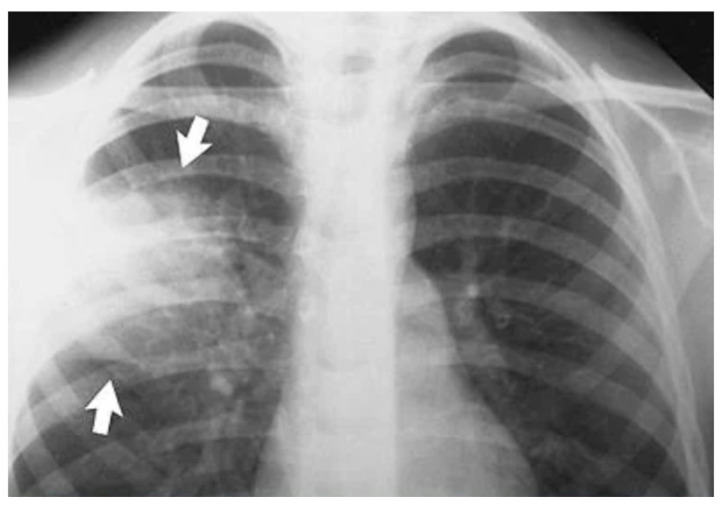
Pneumonia affected Lung. Arrows indicate the target area that is to be predicted.

Accurate and early detection of pneumonia is crucial for timely medical intervention and improved patient outcomes. Radiological imaging, such as chest X-rays and computed tomography (CT) scans, is widely used for diagnosing pneumonia [7]. The interpretation of these images, however, can be time-consuming and subjective, leading to the need for automated solutions. A chest X-ray showing an area of lung inflammation indicating the presence of pneumonia is shown in Figure 1.

Detecting pneumonia in chest X-ray images poses several challenges, and deep learning-based models [8] can be instrumental in addressing these challenges. The challenges are:

- Variability in Image Quality: Chest X-ray image quality can vary significantly, making it challenging to identify subtle abnormalities.
- Overlapping Features: Pneumonia patterns may overlap with other lung conditions, leading to misinterpretations.
- Subjectivity: Interpretation of X-rays is subjective and relies on radiologist expertise.
- Size and Location of Infection: The size and location of pneumonia can affect its visibility in X-rays.
- Co-occurring Conditions: Patients with pneumonia may have co-occurring conditions that complicate interpretation.
- Children and Elderly Patients: Detecting pneumonia in children and the elderly can be challenging due to anatomical and age-related differences.
- Evolution of Infections: Pneumonia can evolve rapidly, and X-ray findings may change.
- Atypical Presentations: Pneumonia may present atypically, deviating from typical radiographic patterns.
- Data Imbalance: Imbalanced datasets can lead to biases in model performance.

To address the above-said challenges, deep learning models are appropriate and have shown promising results in automating pneumonia detection from chest X-ray images. These models can learn complex image features and patterns, making them valuable tools for assisting radiologists and improving accuracy in pneumonia detection. However, it is essential to continuously validate and fine-tune deep learning models using a diverse and representative dataset to address the challenges specific to pneumonia detection. 

### 1.2. Artificial Intelligence-Based Models for Disease Diagnosis

Artificial intelligence (AI)-based deep learning models have shown remarkable performance in healthcare dataset diagnosis, particularly in medical image classification tasks such as diagnosing pneumonia, cancer, and other diseases. These models leverage deep learning techniques, which involve training neural networks with large amounts of labeled data to automatically learn features and patterns from the input data. In the context of healthcare dataset diagnosis, deep learning models collect the relevant medical data, such as images, patient records, or genomic data and preprocess them to ensure consistency and quality. This may involve tasks such as image normalization, noise reduction, and data augmentation to increase the diversity of the dataset and improve model generalization. Researchers select an appropriate deep learning architecture based on the nature of the medical data and the specific diagnosis task. Convolutional Neural Networks (CNNs) are commonly used for medical image classification tasks due to their ability to capture spatial hierarchies of features in images [8,9]. 

Recurrent Neural Networks (RNNs) or transformer-based models may be used for sequential data such as patient records or time-series data. The selected deep learning model is trained on the labeled dataset using an optimization algorithm such as stochastic gradient descent (SGD) or Adam. During training, the model learns to map input data to the correct diagnosis labels by adjusting its internal parameters (weights and biases) iteratively. The trained model is evaluated using a separate validation dataset to assess its performance metrics such as accuracy, precision, recall, and F1-score. Cross-validation techniques may also be employed to ensure the robustness of the model. Once the model has been trained and validated, it can be deployed in clinical settings for real-world diagnosis tasks. Integration with existing healthcare systems may involve considerations such as data privacy, regulatory compliance, and user interface design. Continuous monitoring of the model’s performance is essential to detect any drift or degradation in performance over time. Periodic updates and retraining may be necessary to adapt to changes in the dataset distribution or clinical guidelines. 

Overall, AI-based deep learning models offer significant promise in healthcare dataset diagnosis by enabling more accurate, efficient, and scalable solutions for disease detection and patient care. However, challenges such as data scarcity, model interpretability, and regulatory approval remain important considerations in the development and deployment of these models in clinical practice. Deep learning models learn to adapt to variations in image quality by being exposed to a diverse range of images [9] during training. Data augmentation techniques can also be used to simulate different image qualities. These models can be trained on a large and diverse dataset that includes various lung conditions. They can learn to distinguish between pneumonia and other conditions by capturing subtle differences in image features. Deep learning models can provide an objective assessment by consistently analyzing images based on learned patterns. They can serve as a second opinion or assist radiologists in their diagnoses and models can be trained to detect pneumonia regardless of its size or location. CNNs are particularly well-suited for capturing spatial patterns [9].

Deep learning has become a leading approach in various applications, particularly in representation learning. This methodology employs complex multi-layer neural network architectures to automatically learn data representations by transforming input information into hierarchical abstractions. In the realm of image pattern recognition, deep convolutional neural networks (DCNNs) stand out as the most widely utilized deep learning networks. DCNNs, when provided with a sufficiently large training set, autonomously extract pertinent features from the samples through iterative weight adjustments using backpropagation. This iterative learning process allows DCNNs to uncover feature representations without the need for manually designed input features. With proper training for a diverse and representative dataset, the hand-engineered features are outperformed by the DCNN features in terms of both invariance and selectivity [8]. The automated nature of deep learning enables the analysis of thousands or even millions of cases, surpassing the capacity of human experts to perceive and memorize such a vast amount of information in their lifetime. This attribute makes deep learning robust to the broad spectrum of feature variations among different classes, provided the training set is sufficiently large and diverse for comprehensive analysis [9].

The roots of CNNs can be traced back to non-cognition proposed by Fukushima et al. in the early 1980s [10]. In 1990, LeCun achieved a milestone by training a CNN using backpropagation for the classification of handwritten digit patterns [11]. The application of CNNs expanded to various domains in the early 1990s, including object detection, character recognition, and facial recognition. Schultheiss et al. [12] proposed a method to create synthetic thorax radiographs containing realistic nodules derived from CT scans with perfect ground truth knowledge. They assessed the detection capabilities of nine radiologists and two convolutional neural networks through a reader study. Nodules were artificially inserted into CT volumes, and synthetic radiographs were generated by projecting the volume forward and it comprehensively evaluated computer-aided diagnosis (CAD) systems, radiologists’ performance, and accurate ground-truth labels for nodules from synthetic data. Radiographs used for training U-Net and RetinaNet networks were generated from a public dataset comprising 855 CT scans. For the reader study, 201 radiographs were created from 21 nodule-free CT scans, with varying nodule positions, sizes, and counts. The most effective CAD system detected 268 true positives, with 66 false positives and 102 false negatives. Weighted alternative free response operating characteristic figure-of-merits (wAFROC FOM) for the radiologists ranged from 0.54 to 0.87, while the best-performing CNN achieved a value of 0.81 (CI 0.75–0.87). 

Bengs et al. conducted a systematic comparison of leading object detection algorithms for lung nodule detection, focusing on addressing the challenge of class imbalance. The authors have used data augmentation techniques and transfer learning to enhance performance. By combining insights from this analysis and leveraging multiple architectures, they have achieved a better performance in lung nodule detection. This model was validated by detection track of the Node21 competition [13].

The authors of the research presented in [14] introduced an adaptive transfer learning Deep Convolutional Neural Network (DCNN) for segmenting breast mammogram images containing calcifications, aimed at facilitating early breast cancer diagnosis. They implemented filtering techniques in the region of interest images to enhance image quality by removing artifacts and noise. Through systematic experimentation, they optimized key training parameters such as epoch and batch size to maximize performance. Furthermore, the study compared the performance of the proposed fine-tuned hyperparameter of ResNet50 with other architectures including ResNet34, VGG16, and AlexNet, using confusion matrices for evaluation. Results indicated that the proposed ResNet50 achieved the highest accuracy at 97.58%, followed by ResNet34 with 97.35%, VGG16 with 96.97%, and AlexNet with 83.06%. 

An AI model designed by Pesapane, F et al. [15] for localizing and characterizing microcalcifications, leveraging annotations from three expert radiologists based on histology-derived ground truth. The dataset was divided into training, validation, and testing sets. AlexNet, ResNet18, and ResNet34 architectures were trained and assessed using receiver operating characteristic area under the curve (AUC), sensitivity, and specificity as specific metrics. An evaluation was conducted on the test set, comprising 10% of the total dataset, which encompassed mammograms from 1000 patients aged 21–73 years, totaling 1986 images, with 389 malignant and 611 benign groups of microcalcifications. AlexNet demonstrated a better performance, achieving a sensitivity of 0.98, specificity of 0.89, and AUC of 0.98 for microcalcifications detection, and a sensitivity of 0.85, specificity of 0.89, and AUC of 0.94 for microcalcifications classification. 

The endeavor to detect and diagnose pneumonia encompasses a multifaceted approach, integrating diverse ML and DL methodologies. These methodologies are systematically explored using relevant input datasets, yielding valuable insights crucial for informed healthcare decision-making. Consequently, they serve as the fundamental building blocks for the development of robust pneumonia detection and diagnosis strategies [16,17,18,19].

An active learning approach employed an example re-weighting strategy. In this method, machine-annotated samples are weighted based on similarity of their gradient descent directions to expert-annotated data, and gradient magnitude of the last layer of the deep model. This active learning strategy incorporated a query function to select reliable and informative samples from machine-annotated batch data produced by a noisy data. Validation of clinical COVID-19 CT benchmark data demonstrated that this model improved performance in pneumonia infection segmentation compared to the conventional methods [20]. The VGG16 model [21] was utilized for detecting and classifying pneumonia across two chest X-ray image datasets. The VGG16 model integrated with Neural Networks achieved an accuracy of 92.15%, recall of 0.9308, precision of 0.9428, and F1-Score of 0.937 on the first dataset. Subsequently, experiments were conducted on another dataset comprising 6436 images encompassing pneumonia, normal, and COVID-19 cases. Results from the second dataset yielded an accuracy, recall, precision, and F1-score of 95.4%, 0.954, 0.954, and 0.954, respectively. Comparative analysis revealed that VGG16 performed better than the other models across both datasets.

The noteworthy advancements achieved by deep learning in various pattern recognition applications have sparked considerable enthusiasm and raised hopes for its potential to transform healthcare. Initial research applying deep learning to tasks such as lesion detection or classification has shown promising results, often surpassing conventional methods and, in some instances, even rivaling the performance of radiologists in specific diagnostic tasks [22]. Consequently, deep-learning-based medical image analysis has been increasingly integrated into computer-aided diagnosis (CAD) systems, providing valuable decision support to clinicians and improving the accuracy and efficiency of diagnostic and treatment procedures. The authors have extensively investigated the essential steps required to develop robust deep-learning-based CAD tools and seamlessly integrate them into clinical workflows, thereby contributing to enhanced patient care in healthcare settings [23]. Classifiers can incorporate techniques such as oversampling, under sampling, or using loss functions that weigh rare classes more heavily can mitigate imbalanced data issues. CNNs have shown promising results in automating pneumonia detection from chest X-ray images. LeNet, a classic CNN architecture, has been used in various image classification tasks [24]. In this study, we propose a concatenated modified LeNet classifier that leverages the power of deep learning to accurately classify pneumonia images. The concatenated approach aims to advance the model’s performance by concentrating on different regions of the image, thus reducing the false positive rate and increasing accuracy.

## 2. Literature Survey

In this section, a literature survey on deep learning models for disease diagnosis is discussed which reveals a growing interest in leveraging artificial intelligence techniques to improve medical decision-making. Researchers have explored various deep learning architectures, such as CNNs, RNNs, and transformer-based models, to analyze medical data ranging from imaging scans to electronic health records [8,9,10]. Studies demonstrate the effectiveness of these models in accurately detecting and classifying diseases, including cancer, cardiovascular conditions, and infectious diseases like pneumonia. Additionally, advancements in transfer learning and data augmentation techniques have enabled the development of robust models even with limited labeled data [25,26,27,28,29,30].

### 2.1. Exploring Deep Learning Applications in Medical Imaging

Over the past decade, deep learning has emerged as a potent tool in medical image analysis. Researchers have successfully applied CNNs to various medical imaging tasks, such as tumor detection, organ segmentation, and disease classification. Deep learning models have proven their ability to extract meaningful features from medical images and enhance diagnostic accuracy.

The review of deep neural networks in the realm of medical imaging by Kim et al. [25] utilized Artificial Neural Networks (ANN), and Machine Learning (ML) algorithms. However, limitations arose due to insufficient computing power and limited available training data, leading to issues like overfitting and vanishing gradient problems in deep network training. Advancements in computing power, particularly using graphics processing units (GPUs), and the availability of extensive datasets have revolutionized the field. Deep neural networks have emerged as powerful tools that surpass human and other ML capabilities, especially in tasks related to computer vision and speech recognition. These capabilities have more recently been applied to address healthcare challenges, encompassing applications such as computer-aided detection and diagnosis, disease prediction, image segmentation, and image generation.

While not specific to pneumonia diagnosis, the ResNet architecture introduced in this paper [26] has been widely adopted in medical image analysis, including breast cancer detection. Transfer learning using pre-trained ResNet models has shown promising results in improving classification accuracy.

Although focused on diabetic retinopathy, this paper [27] demonstrated the effectiveness of transfer learning in the medical image analysis domain. The principles discussed can be applied to cancer detection, or any other disease prediction and analysis which highlights the transferability of deep learning models.

This foundational survey [28] has provided a significant insight into the concept of transfer learning and its applications in image classification. While not specific to pneumonia detection, it offered a valuable theoretical background for understanding the principles of transfer learning in medical image analysis.

Researchers designed a novel hybrid optimization algorithm by merging the capabilities of the grasshopper and crow search algorithms [29]. This approach aimed to select optimal features and classify breast masses utilizing a multilayer perceptron (MLP). The proposed model’s performance was compared against various existing optimization algorithms. Evaluations using the MIAS dataset demonstrated that the hybrid approach achieved superior results in several metrics, including classification accuracy (97.1%), sensitivity (98%), and specificity (95.4%).

Researchers have explored the use of multi-layer perceptron (MLP) neural networks for cervical cancer diagnosis. Their model involves tuning the number of hidden layer neurons and utilizing pre-trained deep learning architectures like ResNet-34 and VGG-19 to extract features from the data. Building upon this work, Tan et al. [30] proposed modifications to the classification layers within these convolutional neural networks (CNNs). They then fed the outputs of these modified CNNs, after flattening, into the MLP for final classification. To enhance performance, they trained the CNNs on relevant images using the Adam optimizer. Tested by the Herlev benchmark cervical dataset, this combined approach achieved impressive accuracy, reaching 99.23% and 97.65% for the two classes under investigation.

The authors [31] have reviewed the deep learning methods for image registration, anatomical/cell structures detection, tissue segmentation, computer-aided disease diagnosis and prognosis. They have concluded and shared that deep learning has shed new light on medical image analysis by allowing the discovery of morphological and/or textural patterns in images solely from data. The authors also suggested that ImageNet can find more generalized features in medical images, to enhance the performance. Deep learning models can incorporate data-driven feature representations, and devise a new methodological architecture to reflect the domain-specific knowledge. These models also need to develop algorithmic techniques to efficiently handle images acquired with different scanning protocols to alleviate the need of training modality-specific deep models. They also revealed that it is very difficult to understand and interpret the learned models intuitively.

Balasubramaniam et al. [24] have designed a Modified LeNet architecture (type of CNN) which has been successfully applied to breast cancer data analysis. It demonstrated its ability to extract discriminative features and classify malignant and benign tumors with high accuracy, thereby supporting early detection and diagnosis of breast cancer. LeNet with corrected Rectified Linear Unit (ReLU), a modification of the traditional ReLU activation function, has been found to improve the performance of LeNet in breast cancer data analysis. This has led to more accurate, reliable breast cancer detection and diagnosis and improved patient outcomes. Batch normalization improved the performance and training stability of small and shallow CNN architecture like LeNet. It helped to mitigate the effects of internal covariate shift, which refers to the change in the distribution of network activations during training. This classifier will lessen the overfitting problem and reduce the running time. The designed classifier is evaluated against the benchmarking deep learning models, proving that this has produced a higher recognition rate. The accuracy of the breast image recognition rate was 89.91% and achieved better performance in breast cancer tumor detection [24].

### 2.2. Pneumonia Diagnosis Using Deep Learning

Several studies have explored the use of deep learning for pneumonia diagnosis. These studies have demonstrated the potential of CNNs in distinguishing between normal and pneumonia-infected lungs. Various CNN architectures, including LeNet, VGG, and ResNet, have been adapted and fine-tuned for this task. However, there is room for improving the accuracy and efficiency of these models.

Ibrahim et al. [32] investigated the potential of a deep learning approach for classifying different chest X-ray (CXR) images, including coronavirus disease, non-corona viral pneumonia, pneumococcal pneumonia, and normal CXR scans. The source imagery was compiled from multiple publicly available databases. The study explored various classification scenarios: two-way (e.g., COVID-19 vs. normal), three-way (COVID-19 vs. bacterial pneumonia vs. normal), and four-way (adding non-COVID-19 viral pneumonia). The notable results are:

- Non-COVID-19 viral pneumonia vs. normal: The model demonstrated 94.43% accuracy, 98.19% sensitivity, and 95.78% specificity.
- Bacterial pneumonia vs. normal: The model achieved 91.43% accuracy, 91.94% sensitivity, and 100% specificity.
- COVID-19 vs. normal: Here, the model’s accuracy reached 99.16%, sensitivity 97.44%, and specificity 100%.
- COVID-19 vs. non-COVID-19 viral pneumonia: Accuracy was 99.62%, sensitivity 90.63%, and specificity 99.89%.
- Three-way classification: The model displayed 94.00% accuracy, 91.30% sensitivity, and 84.78% specificity.
- Four-way classification: Accuracy was 93.42%, sensitivity 89.18%, and specificity 98.92%.

Overall, the study’s findings suggest the model’s effectiveness in classifying diverse chest X-ray images, potentially aiding in the diagnosis of various respiratory ailments [32].

To assist radiologists and healthcare practitioners, an automated Computer Aided Diagnosis (CAD) system was developed by Kundu et al. [33] for detecting early pneumonia traces. This CAD system used deep transfer learning-based classification to classify chest X-ray images into two classes “Pneumonia” and “Normal”. The researchers proposed an ensemble framework that leverages the outputs from three CNN models: GoogLeNet, ResNet-18, and DenseNet-121. The framework combines the predictions from these models by creating a weighted average, effectively harnessing the collective knowledge of all three networks. The weights assigned to the classifiers are evaluated using various classification metrics and fused using the hyperbolic tangent function. This CAD model was tested against two datasets, namely Kermany and RSNA datasets. For Kermany datasets, an accuracy of 98.81%, sensitivity of 98.8%, 98.82% precision, and f1-score of 98.79% are obtained. For RSNA datasets, an accuracy of 86.8%, sensitivity of 87.02%, 86.89% precision, and f1-score of 86.95% are obtained. However, this ensembled CAD framework failed to produce correct predictions due to an inappropriate feature extraction method and consumed more computational resources.

Kong and Cheng [34] proposed a deep learning approach for pneumonia diagnosis in X-ray images. This method combines an Xception neural network for feature extraction with Long Short-Term Memory (LSTM) for feature selection and classification. The Xception network extracts informative features from the X-ray images, which are then fed into the LSTM. The LSTM analyzes these features and identifies the most relevant ones for pneumonia detection. The researchers addressed the challenge of imbalanced class distribution during training by employing a hybrid loss function combining cross-entropy loss with Pearson’s correlation-based feature selection. This optimized approach achieved promising results of 96% accuracy and 99% ROC. These results suggest that the model can potentially assist healthcare professionals in diagnosing childhood pneumonia.

### 2.3. Concatenated Classifiers in Medical Imaging

The concept of concatenated classifiers has been widely used in medical imaging. These concatenated models typically consist of multiple stages, each designed to focus on different aspects or regions of an image. By gradually eliminating non-relevant regions, concatenated classifiers can improve overall accuracy and reduce false positives. This approach has been used in tasks like face detection and more recently in medical image analysis [35].

This thesis [36] explored the application of machine learning and image analysis techniques to develop accurate and reliable diagnostic models for medical images. The goal was to assist medical professionals in addressing challenges associated with image interpretation. Due to the complexity of image data distributions, a classifier ensemble approach using the random subspace method was introduced for microscopic image classification. Multi-layer perceptrons served as the foundation for this ensemble model. The researcher investigated various feature extraction methods to find the most suitable representations for microscopic images. To enhance classification reliability in bio-medical images, a cascade classification system was designed. It employed two serially connected, random subspace-based classifier ensembles. The first stage utilized support vector machine base classifiers, while the second incorporated neural networks. Images that did not meet a confidence threshold in the initial stage were escalated to the next for additional analysis. The cascade system’s evaluation on breast cancer biopsy images and UCI datasets demonstrated high classification accuracy and reliability, with minimal image rejections. To address the common issue of imbalanced medical datasets, another ensemble classifier was proposed, employing Kernel Principal Components as base units trained on diverse image features. This method yielded promising results on medical image datasets [36].

Shadi et al. [37] explored various deep learning algorithms for detecting pneumonia in chest X-ray images. Their work included Enhanced CNN, VGG-19, ResNet-50, and a fine-tuned ResNet-50 model. These models were trained in a large dataset compiled from Kaggle and were expanded for this specific study. The dataset encompassed 5863 images divided into training, testing, and validation sets, reflecting real-world data generation scenarios. The experiments yielded notable results: ResNet-50 achieved an accuracy of 82.8%, while the Enhanced CNN outperformed, attaining the highest accuracy of 92.4%, establishing its superiority in this evaluation. The results [37] highlighted the potential of these models in detecting pneumonia progression. This could lead to more accurate diagnoses and timely treatment for patients. Enhanced CNN and fine-tuned ResNet-50 identified pneumonia after proper calibration.

Chun-Fu Yeh et al. [38] introduced a comprehensive screening platform for detecting COVID-19 pneumonia using artificial intelligence. Their AI-based system utilized chest X-ray (CXR) images to predict COVID-19 infection in patients. However, due to the relatively small public collection of CXR images, training a deep neural network (DNN) for accurate predictions posed a challenge. In response, the researchers devised a concatenated learning strategy to enhance both sensitivity and specificity of the DNN classification model. This involved leveraging a sizable dataset of non-COVID-19 pneumonia CXR images to generalize and improve the original model through a concatenated learning approach. The resulting screening system demonstrated effective classification performance on an expanded dataset, which included newly added COVID-19 CXR images.

Karar et al. [39] introduced a novel cascaded deep learning framework aimed at enhancing the effectiveness of computer-aided diagnosis (CAD) systems in the detection of COVID-19 and pneumonia within X-ray imagery. Their approach breaks down complex multi-label X-ray classification into a sequence of binary classifiers tailored to identify specific health conditions. This mirrors real-world diagnostic practices and facilitates the identification of potential illnesses. The flexibility of the cascaded structure enables the concurrent deployment of various fine-tuned deep learning models, maximizing the accuracy in identifying positive cases. The study employed eleven pre-trained CNN, encompassing models like Visual Geometry Group Network (VGG) and Residual Neural Network (ResNet). Comprehensive testing on a public X-ray dataset containing normal and diseased cases validated the model’s performance. Notably, VGG16, ResNet50V2, and Dense Neural Network (DenseNet169) exhibited superior detection accuracy for COVID-19, viral (non-COVID-19) pneumonia, and bacterial pneumonia images, respectively [39].

Optical coherence tomography (OCT) image classification relied on classical CNNs, which, although effective, are criticized for disregarding positional relations in pooling layers [40]. To address this limitation, we explored the use of capsule networks for OCT image classification, leveraging their ability to learn positional information from images. Our study aimed to enhance classification accuracy by replacing CNNs with capsule networks. We curated a training dataset comprising 83,484 OCT images and a test dataset of 1000 images. The training dataset consisted of 37,205 images with choroidal neovascularization (CNV), 11,348 with diabetic macular edema (DME), 8616 with drusen, and 26,315 normal images. The test dataset included 250 images from each category. The model, based on a capsule network, was developed to improve classification accuracy. It underwent training using the training dataset and was subsequently evaluated using the test dataset. Our approach yielded remarkable results, achieving an accuracy of 99.6% in OCT image classification. This accuracy surpasses existing methods in the literature by 3.2 percentage points, highlighting the efficacy of capsule networks in this domain.

The advent of data analytics has revolutionized the healthcare industry, offering invaluable tools for disease prediction through insightful analysis into patient histories [41]. By leveraging predictive modeling, clinicians can make informed decisions with greater accuracy when diagnosing diseases. This paper undertakes a comparison between the J48 and naive Bayes classification techniques, aiming to optimize algorithmic efficiency and accuracy using Thyroid datasets [41]. The results indicate that the decision tree J48 emerged as the superior classifier, boasting an accuracy of 81.94%. In contrast, the Naive Bayes classifier achieved an accuracy of 51.77%. These findings underscore the efficacy of the J48 technique, which outperformed Naive Bayes in terms of classification accuracy.

In the precise diagnosis of interstitial lung disease (ILD), achieving consensus across radiological, pathological, and clinical observations is essential. Mei et al. [42] have used RadImageNet pre-trained models which are a combination of Deep Neural Networks (DNN) and transformer model based approach. RadImageNet models integrate multimodal data to diagnose five ILD types and forecast a patient’s 3-year survival rate. RadImageNet model aided clinicians in diagnosing and categorizing ILD patients, while dynamically predicting disease progression and prognosis. Management of ILD also requires thorough follow-up with computed tomography (CT) studies and lung function tests to assess disease progression, severity, and response to treatment. However, accurate classification of ILD subtypes can be challenging, especially for those not accustomed to reading chest CTs regularly. Thus, dynamic models to predict patient survival rates based on longitudinal data are challenging to create due to disease complexity, variation, and irregular visit intervals.

### 2.4. Challenges and Opportunities

In this paper, we proposed a concatenated modified LeNet classifier for pneumonia image classification. Our approach is to address some of the challenges in pneumonia diagnosis like lack of accuracy, interpretation, and collaboration with HealthCare practitioners. This concatenated model has improved accuracy, and ultimately contributed to more effective patient care. We present the architecture, training process, and evaluate the model’s performance using a comprehensive dataset, demonstrating its potential as a valuable tool in the diagnosis of pneumonia from medical images.

## 3. Proposed Concatenated Modified LeNet-5 Model

**Figure 2:**
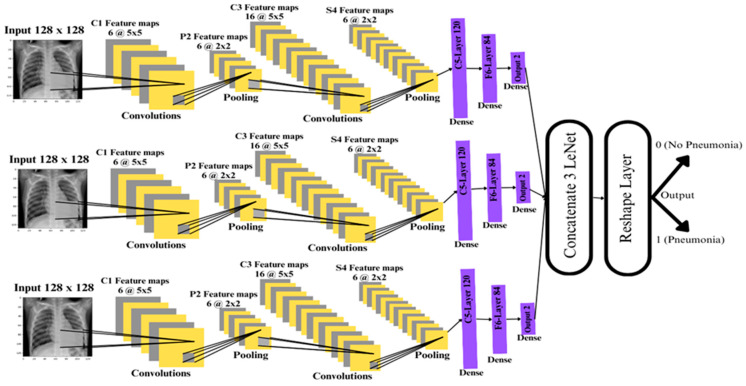
Proposed Concatenated LeNet-5 Model.

The concatenated LeNet-5 model, shown in Figure 2, consists of three identical LeNet-5 models, each processing the input data through a series of convolutional and fully connected layers. The input layer at the bottom of the architecture accepts data with a shape of (128, 128, 1), representing grayscale images of size 128 × 128 pixels. A line connects this input layer to each of the concatenated LeNet-5 model, indicating that the same input is fed into each model.

Each LeNet-5 model follows a consistent architecture. The model begins with a convolutional layer with 6 filters, a kernel size of (5, 5), ReLU activation with a same spatial dimensions as the input ensures that the output feature map has the same spatial dimensions to propagate gradients efficiently. This is followed by an average pooling layer with a pooling size of (2, 2). Subsequently, another convolutional layer is applied with 16 filters, a kernel size of (5, 5), and ReLU activation, followed by another average pooling layer with a pooling size of (2, 2). A flattening layer is then employed to prepare the output for the subsequent dense layers.

After flattening, the concatenated LeNet-5 model incorporates two fully connected layers, with the first 120 neurons and ReLU activation, and the second with 84 neurons and ReLU activation. The final output layer of each LeNet-5 model consists of 2 neurons with softmax activation, enabling binary classification. This entire sequence of layers is repeated three times, once for each concatenated model.

The outputs of the three LeNet-5 models are concatenated using a concatenate layer, creating a unified representation of the intermediate outputs. This concatenated output is then reshaped using a Reshape layer to have dimensions (nets, 2), where ‘nets’ represents the number of concatenated models. Finally, the output layer at the top of the architecture produces the final output with a shape of (None, 2), indicating the binary classification outcome.

The sequence of input, processing and output layer details are presented below.

- Input Layer: At the bottom of the diagram, a layer representing the input data with the shape (128, 128, 1).
- LeNet-5 Model (Repeated Three Times): For each Concatenated model, the following blocks, repeated three times (because nets = 3 in the proposed model):
  ➢ Convolutional Layer: 6 filters, kernel size (5, 5), ReLU activation, with Average Pooling (pool size: 2 × 2).
  ➢ Convolutional Layer: 16 filters, kernel size (5, 5), ReLU activation, with Average Pooling (pool size: 2 × 2).
  ➢ Flatten Layer: Flattens the output.
  ➢ Fully Connected Layer: 120 neurons, ReLU activation.
  ➢ Fully Connected Layer: 84 neurons, ReLU activation.
  ➢ Output Layer: 2 neurons with softmax activation.
- Concatenated Input: A line connects the input layer to each concatenated LeNet-5 model, indicating that the same input is fed into each.
- Concatenate Layer: Above the LeNet-5 blocks, there will be a block representing the concatenate layer, combining the outputs of the concatenated models.
- Reshape Layer: Above the concatenate layer, there will be a block representing the Reshape layer, reshaping the concatenated output to have dimensions (nets, 2).
- Output Layer: In the top, there will be the final output layer with shape (None, 2).

Each block in Figure 2 represents a layered architecture with specified configurations, the lines between them indicate the flow of data, and the connections in the concatenated LeNet-5 model.

**Figure 3:**
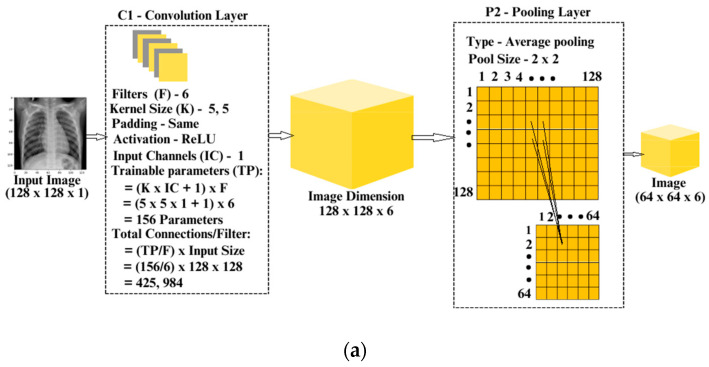

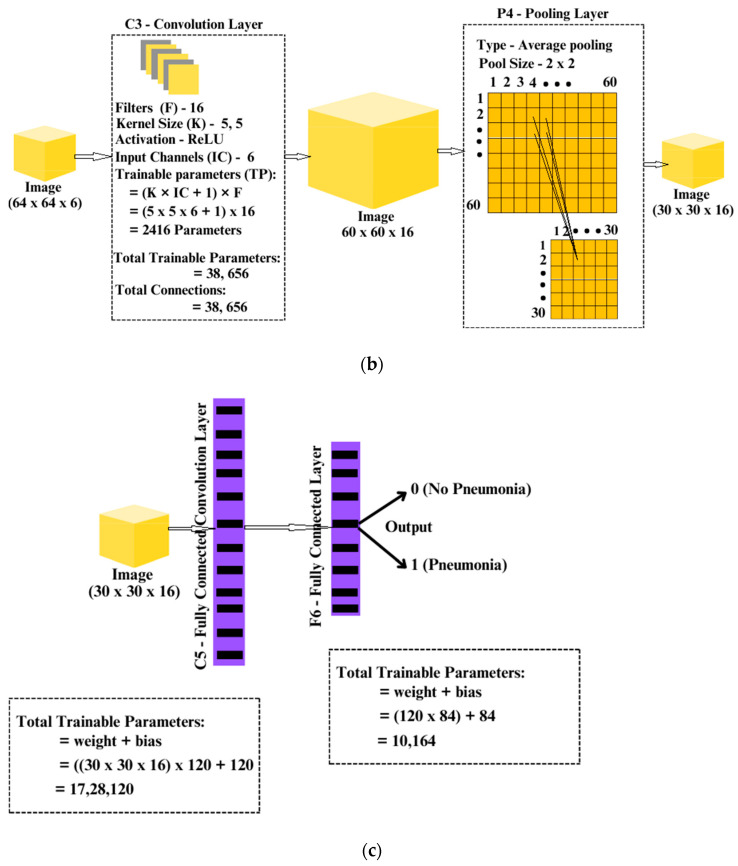
(**a**) Convolution and Pooling Layers of the Concatenated LeNet—Layer 1 and 2. (**b**) Convolution and Pooling Layers of the Concatenated LeNet—Layer 3 and 4. (**c**) Convolution and Pooling Layers of the Concatenated LeNet—Layer 5.

Detailed information about the layers, and the connections of the concatenated LeNet model is presented in Figure 3a–c.

**Figure 4:**
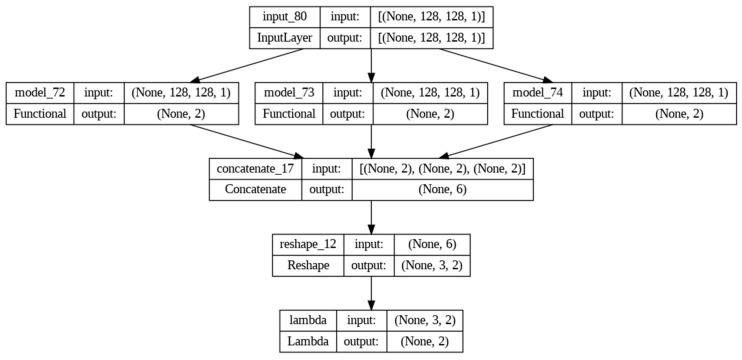
Design and Implementation Summary of the Concatenated LeNet Model.

The model is compiled using the Adam optimizer and categorical crossentropy loss, and a summary of the architecture is displayed in Figure 4.

## 4. Results

### 4.1. Dataset Description

The Chest X-ray Images Dataset is publicly available [43]. All the images are in PNG format and categorized into two classes, which are pneumonia (1), and non-pneumonia (0) indicating a resolution of 128 × 128 pixels. In the dataset, total number of images are 5856 in which non-pneumonia images are 1583 and pneumonia images 4273. In training, 4684 images of both pneumonia and non-pneumonia are used. For validation and testing, 1152 and 20 images are used, respectively.

### 4.2. Experimentation Details

**Figure 5:**
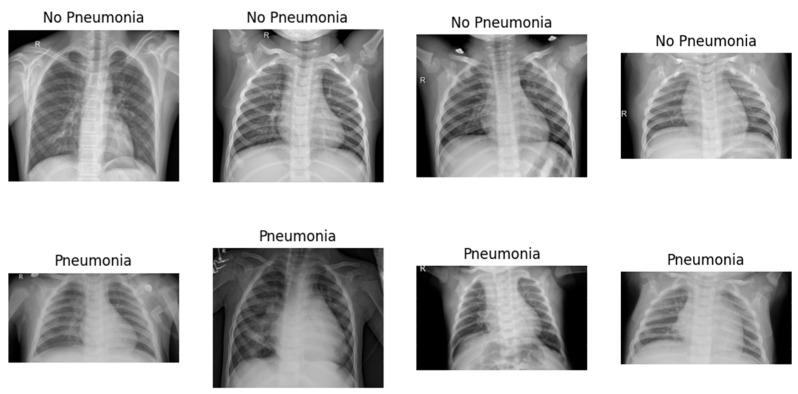
Sample Pneumonia and Non-Pneumonia chest X-ray Images.

In our experiments, we utilize Google Colab, a cloud-based platform offering Jupyter notebook functionality. This platform grants access to image sources through its connection to Google Drive. For accelerated training, we leverage T4 class GPUs offered by Google Colab. The deep learning models are built using Keras 2.6.0, a user-friendly library for constructing neural networks, which seamlessly integrates with TensorFlow 2.14.0, a powerful open-source framework for machine learning. This combined environment, accessible through Google Colab, allows for effortless library import and code execution within a collaborative setting. This integration simplifies model development while ensuring optimal utilization of the available GPU resources. The implementation of the concatenated model with the input images carried out using Python 3.10.11. The confusion matrix serves as a vital tool in evaluating the efficacy of a concatenated modified LeNet-5 model for image recognition, specifically in distinguishing between pneumonia and non-pneumonia images and the sample classification is shown in Figure 5. This matrix, typically presented as a 2 × 2 table for binary classification, encapsulates the counts of true positives (TP), true negatives (TN), false positives (FP), and false negatives (FN) based on the model’s predictions on a test dataset.

In the domain of pneumonia classification, we employ the concatenated modified LeNet-5 model. The evaluation of this model includes comprehensive performance metrics, shedding light on its efficacy in accurately classifying pneumonia cases. These metrics not only gauge the model’s accuracy but also provide insights into its precision, recall, and F1 score, allowing a nuanced understanding of its classification capabilities. The implications of these performance metrics extend beyond numerical values, offering crucial insights into the model’s strengths and areas for improvement. This analytical approach ensures a thorough assessment of the concatenated modified LeNet-5 model’s proficiency in pneumonia classification, providing valuable information for further refinement and optimization.

1. Sensitivity (True Positive Rate): This metric highlights the model’s ability to correctly identify images with pneumonia. It is calculated as TP divided by the sum of TP and FN. A higher sensitivity indicates a stronger capability to catch actual cases of pneumonia.
2. Specificity (True Negative Rate): Specificity gauges the model’s proficiency in correctly identifying non-pneumonia images. It is computed as TN divided by the sum of TN and FP. A higher specificity implies a reduced likelihood of misclassifying non-pneumonia instances.
3. Precision: Defined as the ratio of true positive predictions to the total instances predicted as positive (TP/(TP + FP)), precision measures the model’s ability to accurately identify pneumonia cases among all instances it predicts as positive. In the medical diagnosis context, precision becomes crucial as it reflects the proportion of predicted positive cases that are indeed true positives. High precision indicates that when the model predicts pneumonia, it is likely to be correct, minimizing the risk of false alarms in clinical settings.
4. F1-score: The F1-score combines precision and recall, striking a balance between the two metrics. It is the harmonic mean of precision and recall, providing a comprehensive measure of a model’s performance by considering both false positives and false negatives. The F1-score is particularly valuable in medical diagnosis, where achieving equilibrium between correctly identifying pneumonia cases (recall) and ensuring those predictions are accurate (precision) is paramount. A higher F1-score signifies a model that excels in both precision and recall, crucial for reliable and trustworthy pneumonia detection. Additionally, support indicates the number of actual occurrences of each class, providing context to the precision and recall values and aiding in the interpretation of the model’s performance across different class sizes.

**Figure 6:**
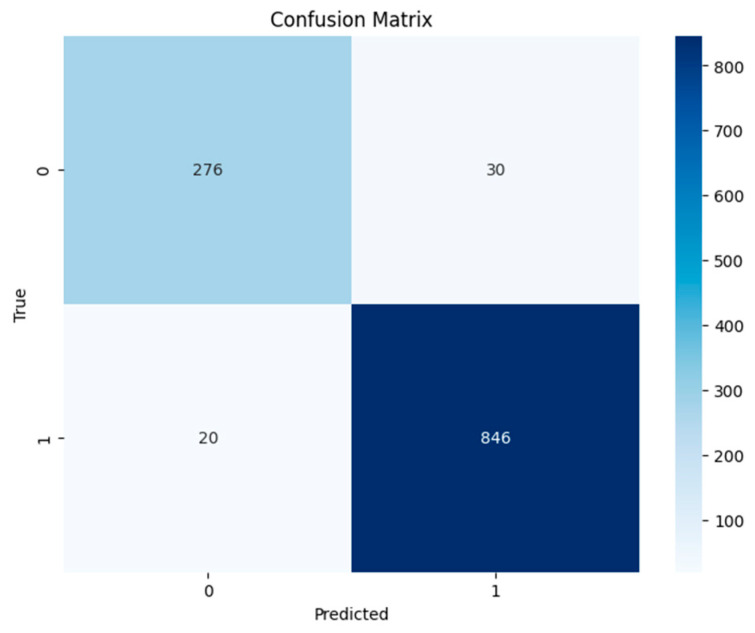
Confusion matrix for the Concatenated Modified LeNet Model.

**Figure 7:**
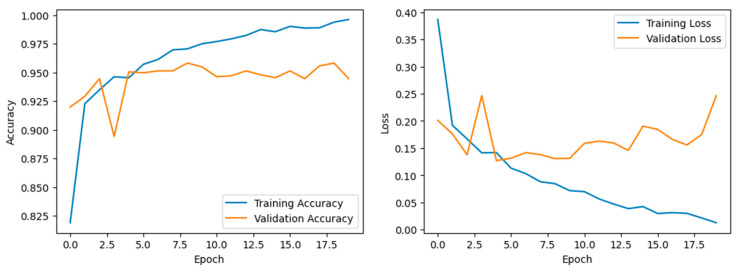
Accuracy and Validation losses of the Concatenated LeNet Model.

In a medical diagnosis scenario, understanding the support for pneumonia and non-pneumonia classes helps to contextualize the model’s generalization to real-world prevalence rates, contributing to a more nuanced assessment of its effectiveness in clinical applications. The confusion matrix offered a detailed insight into how well the concatenated modified LeNet-5 architecture performs in classifying pneumonia cases, which is shown in Figure 6. Table 1 and Figure 7 illustrate the model’s efficacy in image recognition. It accurately distinguishes between 293 non-pneumonia cases and 824 pneumonia cases, showcasing high true negative and true positive values, respectively. However, it does show 22 false positives, indicating instances where non-pneumonia cases were mistakenly identified as pneumonia. Additionally, there are 33 false negatives, where the model incorrectly categorized pneumonia cases as non-pneumonia.

The confusion matrix is a powerful tool for assessing the performance of a classifier, offering insights that go beyond simple accuracy metrics and providing a more detailed picture of the model’s strengths and weaknesses. The concatenated LeNet5 model, as described, is a unique architecture that involves the combination of three identical LeNet-5 models. Each LeNet-5 model processes the input data independently through a series of convolutional and fully connected layers. The key features of this model are its use of three parallel LeNet-5 structures and the concatenation of their outputs. The use of three identical LeNet-5 models in parallel can be seen as a form of ensemble learning. Ensemble methods combine multiple models to improve overall performance, and in this case, the concatenation of outputs may capture diverse representations from the input data.

- Feature Diversity: Each LeNet-5 model processes the input data independently, capturing different aspects and features. Concatenating these outputs likely results in a more comprehensive representation of the input data, contributing to improved accuracy.
- Parameter Sharing: Since the LeNet-5 models are identical, they share the same set of parameters. This can help in reducing the overall model complexity while still benefiting from the parallel processing of multiple instances.
- Effective Representation Learning: The architecture’s ability to achieve higher accuracy on both training and testing datasets suggests that it effectively learns and generalizes representations from the input data, outperforming other architectures like Modified LeNet, ResNet 50, and AlexNet.
- Insight into Class Imbalances: In situations where there is a class imbalance (significant difference in the number of instances between classes), a confusion matrix helps identify how well the model performs for each class.
- Model Comparison: When comparing multiple models, a confusion matrix facilitates a side-by-side evaluation of their performance, enabling stakeholders to make informed decisions about which model is better suited for a particular task.
- Diagnostic Information: The confusion matrix is particularly useful in medical and diagnostic applications, providing information on the model’s ability to correctly identify positive (disease presence) and negative (disease absence) cases.

The concatenated LeNet-5 model employs a parallel processing approach, leveraging three identical LeNet-5 structures to capture diverse features from the input data. The concatenation of these outputs appears to enhance the model’s representation learning, leading to superior training and testing accuracies of 98% and 95%, respectively, compared to other specified architectures. Modified concatenated LeNet-5 architecture demonstrated significant improvements in image recognition tasks, particularly in distinguishing between pneumonia and non-pneumonia cases, even when dealing with large datasets which can be inferred from Table 2.

**Figure 8:**
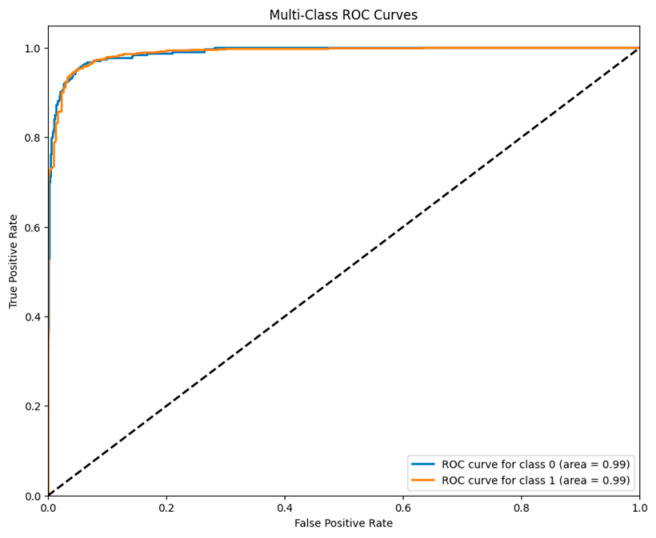
ROC curve for the Concatenated Model.

**Figure 9:**
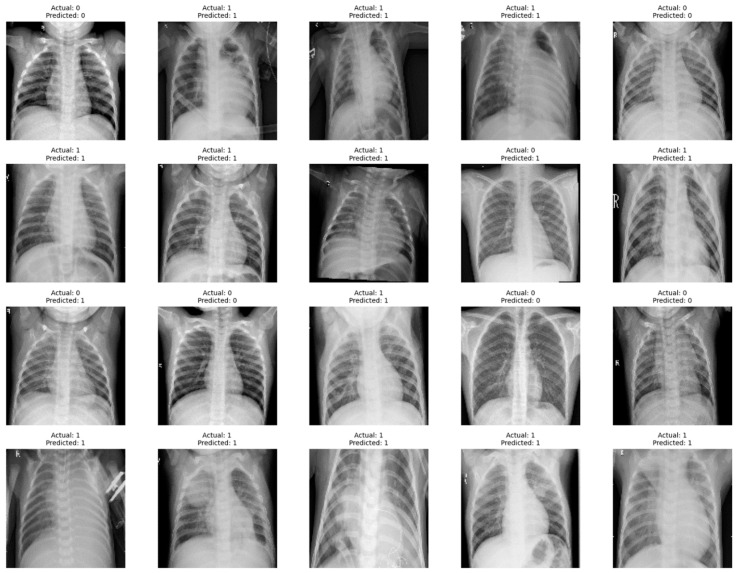
Predicted by the Proposed Concatenated Modified LeNet Classifier.

Figure 8 shows the implications of a multiclass ROC curve drawn based on a true positive (TP) versus false positive (FP) for a concatenated M=modified LeNet-5 model on a pneumonia dataset and its predictive power is depicted in Figure 9. The area under the ROC curve (AUC) of the multiclass ROC curve can provide an overall measure of how well the concatenated modified LeNet-5 model discriminated between pneumonia and non-pneumonia classes. Here, AUC of 0.99 is obtained which is higher AUC value. From this, it is inferred that the model has shown the better overall performance. The true positive rate (TPR), or sensitivity metric, indicated the ability of the model to correctly identify instances of pneumonia. A high sensitivity is crucial for a medical diagnosis task as it minimized the number of false negatives, ensuring that actual cases of pneumonia are not missed.

The false positive rate (FPR), or specificity metric, reflected the rate of misclassification of non-pneumonia instances as pneumonia. In a medical context, minimizing false positives is essential to avoid unnecessary treatments or interventions. The model’s performance is crucial for clinical decision support, particularly in accurately identifying pneumonia cases, as it directly impacts patient care. It is essential to emphasize that incorporating domain experts’ knowledge is key when interpreting the model’s results in medical applications. This collaboration will ensure that the model’s predictions align with clinical expectations, guiding appropriate patient care, medication decisions, and therapy details. The involvement of healthcare professionals is paramount to the successful integration of the model into the clinical decision-making process.

**Figure 10:**
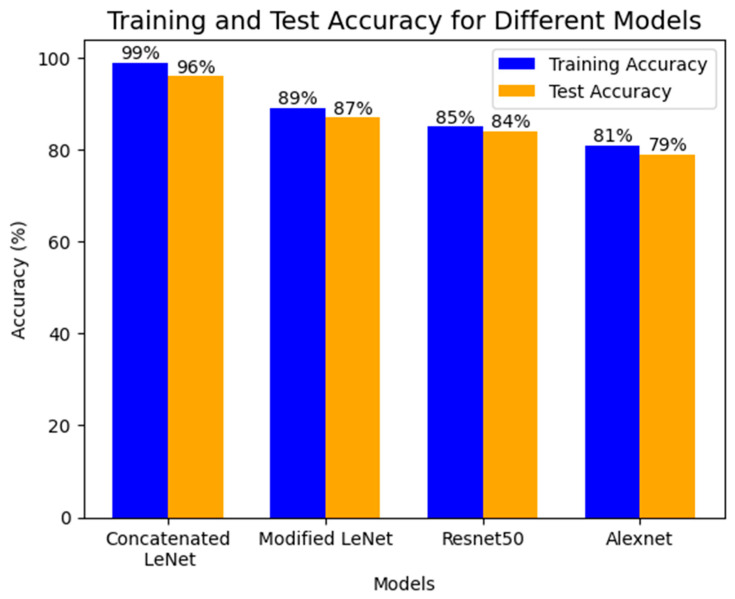
Comparison of Training and Test Accuracy values.

The design of the concatenated LeNet-5 model, which achieved higher training and testing accuracies compared to classifiers like modified LeNet, ResNet 50, and AlexNet, suggested several benefits that contribute to its superior performance, which are shown in Table 2 and Table 3 and Figure 10.

- Ensemble Learning: The use of three identical LeNet-5 models in parallel introduced an ensemble learning strategy. Ensemble methods combine multiple models to improve overall performance by capturing diverse patterns and representations. In the concatenated LeNet-5 model, the parallel processing of three identical models enabled the extraction of complementary features from the input data. The subsequent concatenation of these diverse features contributes to a more robust and generalized model.
- Comprehensive Feature Extraction: The concatenated LeNet-5 architecture allowed for a comprehensive extraction of features from the input data. Each LeNet-5 model processed the input independently, capturing different aspects and details. By concatenating these outputs, the model can aggregate a more extensive set of features, enhancing its ability to discriminate between classes and improving accuracy.
- Parameter Sharing: The three LeNet-5 models in the concatenated LeNet-5 architecture are identical, meaning they shared the same set of parameters. Parameter sharing reduced the overall model complexity, making it more efficient and preventing overfitting on the training data. This shared parameterization also facilitates effective learning and generalization, contributing to the model’s high accuracy on both training and testing datasets.
- Reduction of Overfitting: The ensemble nature of the concatenated LeNet-5 model helped mitigate overfitting. Overfitting occurred when a model learnt to perform well on the training data but failed to generalize to unseen data. The diversity introduced by parallel LeNet-5 models and their subsequent combination through concatenation aids in reducing overfitting, leading to better generalization and higher testing accuracy.
- Parallel Processing and Efficiency: The parallel processing of three LeNet-5 models allows for efficient computation, enabling faster training times. This can be particularly advantageous when dealing with large datasets and complex architectures. The efficiency gained through parallel processing contributes to quicker convergence during training, resulting in a model that achieves higher accuracy in a shorter amount of time.
- Effective Learning Representations: The Concatenated LeNet-5 model exceled in learning effective representations of the input data. The combination of parallel processing, ensemble learning, and concatenation of outputs results in a model that captured both low-level and high-level features, contributing to its ability to make accurate predictions.

The concatenated LeNet-5 model achieved higher training and testing accuracies compared to Modified LeNet, ResNet 50, and AlexNet through a combination of ensemble learning, comprehensive feature extraction, parameter sharing, reduction of overfitting, and efficient parallel processing. These design choices collectively contributed to a model that is not only powerful in learning from the training data but also effective in generalizing to unseen test data, leading to impressive accuracy rates.

The success of the concatenated LeNet-5 models in achieving higher precision, recall, F1-score, and support compared to other models signifies its proficiency in both minimizing false positives and false negatives and maximizing overall predictive performance. The concatenated LeNet-5 model accomplished this using three identical LeNet-5 models in parallel, constituting an ensemble learning approach. In the concatenated LeNet-5 model, the ensemble learning strategy aided in creating a more robust and reliable predictor. The three models processed the same input data independently and captured the diverse patterns and representations. The concatenation of the outputs allowed the integration of these features, providing a richer representation of the data. This comprehensive feature extraction is crucial for achieved higher precision, recall, F1 score, and support, as it enabled the model to discern subtle patterns and variations in the input. Parameter sharing promotes efficient learning and generalization, preventing overfitting of the training data. The shared parameters contribute to the model’s ability to discriminate between different classes by capturing common features across instances. This shared knowledge enhanced precision by reducing false positives and recall by minimizing false negatives.

The diversity introduced by the ensemble models, combined with concatenation, reduces the risk of overfitting, and improves the model’s capacity to generalize, positively impacting precision, recall, and the F1 score. The concatenated LeNet-5 model excels in learning effective representations of the input data. By combining the strengths of three LeNet-5 models, the architecture captures both low-level and high-level features, enabling more accurate predictions. This contributes to higher precision and recall, as the model is adept at identifying relevant patterns in the data.

**Figure 11:**
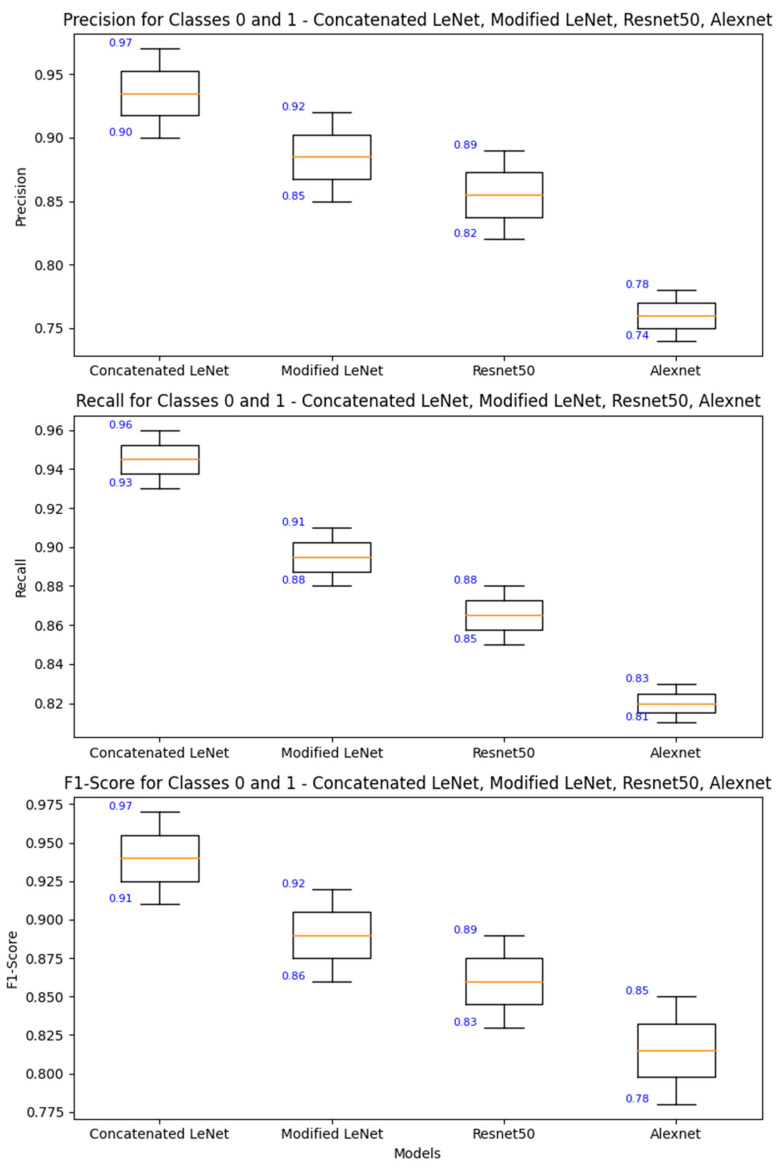
Comparison of Precision, Recall and F1—Scores of the Concatenated and Related Models.

The higher precision, recall, F1 score, and support indicate that the concatenated LeNet-5 model offers a more comprehensive and accurate evaluation of its predictive performance across multiple metrics which is shown in Figure 11 in comparison with other related benchmarked models. This is particularly valuable in real-world scenarios where different aspects of model performance are crucial. The higher precision and recall suggest that the concatenated LeNet-5 model is more confident in its predictions. This is crucial in applications where the consequences of false positives or false negatives are significant, such as in medical diagnoses or fraud detection. The model’s ability to achieve high precision, recall, F1 score, and support implied that it generalized well for unseen data. This is a key consideration in deploying machine learning models in real-world scenarios where robust performance on new, previously unseen instances is essential. In summary, the concatenated LeNet-5 model’s success in achieving higher precision, recall, F1 score, and support is attributed to its ensemble learning strategy, comprehensive feature extraction, parameter sharing, and efficient learning representations. The implications for model analysis include a holistic evaluation, robustness across various metrics, increased confidence in predictions, and strong generalization for unseen data [39].

The advantages of the concatenated deep learning classifiers framework include:

- Improved performance in confirming infected cases
- Flexibility in using different fine-tuned deep learning models
- Ability to handle multi-label classification of X-ray images
- Successful testing and evaluation on a public X-ray image dataset

This framework has the potential to significantly enhanced the accuracy of CAD systems for diagnosing pneumonia diseases.

## 5. Conclusions

The concatenated modified LeNet classifier, when incorporated, corrected rectified linear unit (ReLU) activation functions and batch normalization. A promising solution for accurate pneumonia image classification is presented. The utilization of deep learning techniques enhanced the diagnostic capabilities, facilitating more efficient and timely patient care. The concatenated modified LeNet architecture with corrected ReLU contributes to improved feature extraction, and boosted the model’s discriminative power. The inclusion of batch normalization further enhanced the stability and performance of the classifier, particularly in small and shallow convolutional neural network (CNN) architectures like LeNet. The evaluation of the model on a comprehensive dataset demonstrates a remarkable recognition rate, achieving an accuracy of 96% in pneumonia image classification. This performance is competitive with or even surpassed other relevant deep learning models like those benchmarked in this study. Notably, the classifier addressed common challenges such as overfitting and reducing running time, suggesting its potential as a valuable diagnostic tool in the field of pneumonia diagnosis from medical images. These findings underscore the significance of leveraging advanced deep learning techniques in medical image analysis, particularly for critical health concerns like pneumonia. The proposed concatenated modified LeNet classifier holds promise for practical implementation, offering a reliable and effective means to assist healthcare professionals in the accurate diagnosis of pneumonia, ultimately contributing to improved patient outcomes.

## Figures and Tables

**Table 1:** Classification Report of the proposed LeNet Model.

Class Prediction	Precision	Recall	f1-Score	Support
Non-Pneumonia	0.93	0.90	0.92	306
Pneumonia	0.97	0.98	0.97	866
Accuracy			0.96	1172
weighted avg	0.95	0.94	0.94	1172
weighted avg	0.96	0.96	0.96	1172

**Table 2:** Image Recognition for the Pneumonia and Non-Pneumonia cases.

		Predicted	
		0	1
Actual	0	True Negative 293	False Positive 22
	1	False Negative 33	True Table 2 Positive 824

**Table 3:** Comparison of Training and Testing Accuracy values of different models.

	Training Accuracy	Test Accuracy
Concatenated LeNet	99	96
Modified LeNet	89	87
Resnet50	85	84
Alexnet	81	79

## Data Availability

Data are contained within the article.
